# Fructose, insulin resistance, and metabolic dyslipidemia

**DOI:** 10.1186/1743-7075-2-5

**Published:** 2005-02-21

**Authors:** Heather Basciano, Lisa Federico, Khosrow Adeli

**Affiliations:** 1 Clinical Biochemistry Division, Department of Laboratory Medicine and Pathobiology, Hospital for Sick Children, University of Toronto, Toronto, Ontario, Canada

## Abstract

Obesity and type 2 diabetes are occurring at epidemic rates in the United States and many parts of the world. The "obesity epidemic" appears to have emerged largely from changes in our diet and reduced physical activity. An important but not well-appreciated dietary change has been the substantial increase in the amount of dietary fructose consumption from high intake of sucrose and high fructose corn syrup, a common sweetener used in the food industry. A high flux of fructose to the liver, the main organ capable of metabolizing this simple carbohydrate, perturbs glucose metabolism and glucose uptake pathways, and leads to a significantly enhanced rate of *de novo *lipogenesis and triglyceride (TG) synthesis, driven by the high flux of glycerol and acyl portions of TG molecules from fructose catabolism. These metabolic disturbances appear to underlie the induction of insulin resistance commonly observed with high fructose feeding in both humans and animal models. Fructose-induced insulin resistant states are commonly characterized by a profound metabolic dyslipidemia, which appears to result from hepatic and intestinal overproduction of atherogenic lipoprotein particles. Thus, emerging evidence from recent epidemiological and biochemical studies clearly suggests that the high dietary intake of fructose has rapidly become an important causative factor in the development of the metabolic syndrome. There is an urgent need for increased public awareness of the risks associated with high fructose consumption and greater efforts should be made to curb the supplementation of packaged foods with high fructose additives. The present review will discuss the trends in fructose consumption, the metabolic consequences of increased fructose intake, and the molecular mechanisms leading to fructose-induced lipogenesis, insulin resistance and metabolic dyslipidemia.

## Emerging epidemic of the Metabolic Syndrome

The new millennium has witnessed the emergence of a modern epidemic, the metabolic syndrome, with frightful consequences to the health of humans worldwide. The metabolic syndrome, also referred to as "Diabesity" [1] describes the increasing incidence of diabetes in combination with obesity as a result of changes in human behaviour, available nutrition, and the adoption of more sedentary lifestyles. Obesity and type 2 diabetes are occurring at epidemic rates in the United States [2-4] and developing countries including China [5] and India [6]. From 1935 to 1996, the prevalence of diagnosed type 2 diabetes climbed nearly 765% [7]. The global figures are predicted to rise 46% from 150 million cases in 2000 to 221 million in 2010 [8]. This epidemic of type 2 diabetes is complicated by the fact that it is a multi-factorial disease, frequently associated with a cluster of pathologies including obesity, hypertriglyceridemia, impaired glucose tolerance, and insulin resistance, collectively referred to as the metabolic syndrome (formerly known as syndrome X and insulin resistance syndrome). Although there is no universally accepted definition of the metabolic syndrome, most would agree that the syndrome includes a cluster of common pathologies: obesity, insulin resistance, dyslipidemia, and hypertension. It is present in 25–50% of the United States population [9]. There has been a heightened awareness of the metabolic syndrome and a subsequent increase in clinical attention directed towards prevention, due to its strong association with premature morbidity and mortality [8,10]. In particular, these risk factors predispose the individual to greater risk for developing cardiovascular disease and Type 2 diabetes. Recently, the National Cholesterol Education Panel (NCEP) has officially described and identified a number of these risk factors for cardiovascular diseases [11]. These include: 1) abdominal obesity, 2) elevated TG levels, 3) low high density lipoprotein (HDL)-cholesterol levels, 4) increased blood pressure, and 5) impaired fasting glucose [12]. There is also now consensus that insulin resistance and obesity are actually part of one common pathologic mechanism of the metabolic syndrome [13,14]. Evidence shows that the metabolic syndrome process begins early in life and persistence from childhood to adolescent/adult life produces type 2 diabetes and cardiovascular disease [15,16]. The symptoms of metabolic syndrome are not necessarily manifestations of age, but develop over a predisposed background established at a young age [17,18]. This is a dangerous predisposition, with trends in modern diet and habit likely influencing health and behaviour in increasingly younger populations.

The main driving forces for the increased prevalence of insulin resistance are modern Westernized diets and patterns of eating associated with the dramatic rises in obesity. Insulin resistance is often linked to the macronutrient content in the diet. In the past, diets high in saturated fats have been shown to induce weight gain, insulin resistance, and hyperlipidemia in humans and animals [19-22]. Recent research suggests that a high intake of refined carbohydrates may also increase the risk of insulin resistance [23-26]. In addition, diets specifically high in fructose have been shown to contribute to a metabolic disturbance in animal models resulting in weight gain, hyperlipidemia [27], and hypertension [28].

## Nutritional factors influencing the development of the Metabolic Syndrome

Nutrition represents a lifestyle element that can be controlled, and that can directly influence health; therefore preventative nutrition and weight control should become a main focus of consumers and prepared-food providers [29]. The Westernization of diets, with an increase in availability of high calorie foods certainly contributes to the epidemic of metabolic syndrome. In the past, physicians and scientists have made an association between dietary energy from fat and body fat. A large market has developed for the popularity and promotion of low fat diets. Interestingly, however, the decline in dietary fat consumption has not corresponded to a decrease in obesity – in fact, the opposite trend has emerged [30]. Certainly, diets high in saturated fats have been shown to induce weight gain, insulin resistance, and hyperlipidemia in humans and animals [19-22,31], but the emphasis on fat reductions has had no significant benefits relative to the obesity epidemic. More importantly, the focus on dietary fat is more likely a distraction to more significant causes of metabolic syndrome [30]. If fat is not the culprit in metabolic disorders, then what is? Increasing evidence now suggests that the rise in consumption of carbohydrates, *particularly *refined sugars high in fructose, appears to be at least one very important contributing factor.

## Carbohydrates and the link to the Metabolic Syndrome

The general increases in consumption of calories, and specifically of refined carbohydrates and fructose, is clear and correlates positively with an alarming increases in metabolic syndrome. Can these seemingly harmless nutrients actually be directly associated with metabolic syndrome? Recent studies appear to support this link. In a 2004 study, Gross *et al *examined nutrient consumption in the United States between 1909 and 1997, and discovered there was a significant correlation in the prevalence of diabetes with fat, carbohydrate, corn syrup, and total energy intakes. Most striking was the fact that when total energy intake was accounted for, corn syrup was positively associated with type 2 diabetes, while protein and fat were not [32]. High fructose corn syrups (HFCS) are quite commonly found in soft drinks and juice beverages, and are incorporated into many convenient pre-packaged foods, such as breakfast cereals and baked goods. Fructose consumption has thus largely increased over the past few decades most likely as a result of this increased use of HFCS, which contains between 55–90% fructose. The use of HFCS has increased an alarming 1000% between 1970 and 1990 [33]. In 1970, individual consumption of fructose was only 0.5 lb/year. However, in 1997, this figure rose to an alarming 62.4 lb/year [34]. The *type *of common, general use sweeteners represent as large an impact as the dramatic *increase *in the use of these caloric sweeteners. Between 1909 and 1997, sweetener use increased by 86%; and specifically, corn syrup sweeteners now represent over 20% of total daily carbohydrate intake, at an increase of 2100% [32].

These documented trends have inspired a number of consumption studies and recommendations towards HFCS intake. In 1992, the USDA recommended that only 40 g of extra sugars should be added to a standard 2000 calorie a day diet [35]. The amount of HFCS found in only one 12-oz soft drink equals this total proportion of daily intake. HFCS consumption trends are further exacerbated by the fact that soft drink and fruit juice consumption itself has increased dramatically, adding even more extraneous calories and fructose to the diet. From 1965 to 1996, a food consumption study involving 11 to 18 year olds revealed that total energy and fat intakes were decreasing. There were significant decreases in milk consumption but large increases in the consumption of soft drinks and non-citrus juices [36]. Increasingly, children seem to be choosing mass-produced, 'tasty' artificial juices and sodas over healthier alternatives. In a recent letter to the editor, Jacobson [37] illustrates some important factors that contribute to increased consumption of soft drinks, and the link to obesity; a) Society is constantly bombarded by huge million-dollar advertising campaigns for soft drinks, offered extra-extra-large serving sizes with free refills, and surrounded by ubiquitous access to soft drink vending machines even in schools, and b) children's standard drinks to accompany meals, and especially fast food, have become soft drinks. The increased use of HFCS in soft drinks and food products are thus exacerbated by increased exposure, and consumption of these products. HFCS are the main caloric sweeteners utilized in soft drinks in the United States, with fructose representing over 40% of sweeteners added to prepared foods and beverages [33]. In a study of females aged 12 to 19 years milk intake decreased by 36%, whereas sodas and fruit drink consumption increased to nearly double from the 1970s to the mid 1990s. From 1994 to 1996, it was found that even though intake of soda, juices, tea, and alcoholic beverages remained constant, the steady decrease of milk intake continued [38]. This becomes a major problem, because while these high-calorie beverages are being consumed, calories from the rest of the diet are not subsequently reduced. The reality is that people do not eliminate or reduce their food portions because they drank a can of soda that day. Data indicate that energy from beverages generally does not displace or decrease energy from other foods consumed, leading to energy imbalances [39]. The main diet issues involve a general lack of education and/or understanding of the implications with recent consumption patterns. Despite education programs to prevent obesity and diabetes worldwide, there has been little focus on the reduction of fructose and HFCS in beverages.

## Fructose metabolism

Fructose is readily absorbed and rapidly metabolized by human liver. For thousands of years humans consumed fructose amounting to 16–20 grams per day, largely from fresh fruits. Westernization of diets has resulted in significant increases in added fructose, leading to typical daily consumptions amounting to 85–100 grams of fructose per day. The exposure of the liver to such large quantities of fructose leads to rapid stimulation of lipogenesis and TG accumulation, which in turn contributes to reduced insulin sensitivity and hepatic insulin resistance/glucose intolerance. These negative effects of fructose are the reason that fructose metabolism has gained recent research attention. Interestingly, small catalytic quantities of fructose can have positive effects, and actually decrease the glycemic response to glucose loads, and improve glucose tolerance. These effects are also observed without any changes in insulin responses and non-esterified fatty acid (NEFA) and TG levels [40,41]. In 1976, sugar substitutes such as fructose had been found to offer the 'advantage' of a 'better' utilization in conditions of limited insulin production. Fructose had a smaller influence on serum insulin concentrations than glucose, and no influence on plasma glucose levels. At that time, this evidence was considered to support fructose as a positive treatment for diabetic control [40]. In 1986 HFCS were even proposed as a low-cost substitute for fructose in diabetic management. Based on these early observations, nutritive sweeteners were considered safe by the Food and Drug Administration, although, it has now been found that intakes above 25% of total energy consumed will cause hypertriglyceridemia and gastrointestinal symptoms [42]. Even with the early positive results, researchers noticed accompanying "unfavorable" influences of these so-called diabetic sugars on obesity and weight gain. Certain metabolic differences exist between glucose and fructose, and the results that were once thought favorable, proved exacerbating to insulin resistance and obesity. In a study comparing normal and diabetic patients, glycemic effects of HFCS were compared to glucose. The negative results of HFCS on immunoreactive insulin, glycemic effect, and immunoreactive C-peptide did not support its use as a substitute for glucose in diabetic patients [43].

Unfortunately, one out of every four children in the United States consumes above the recommended 25% of total energy intake from sweeteners [42] and the harmful effects of fructose have been extensively studied in healthy, non-diabetic patients. Studies involving commonly consumed fruit juices showed that natural fructose carbohydrates can alter lipid and protein oxidation biomarkers in the blood, and mediate oxidative stress responses *in vivo *[44]. A comparative study by Raben *et al*. examined overweight men and women who consumed fructose-containing sucrose, as opposed to artificial sweeteners as supplements to their diet. Weight, fat mass, and blood pressure were found to be lower in the artificial sweetener-consuming group compared to the sucrose-consuming group, and the sucrose group did not decrease intake of other nutrients to compensate for their increased calorie consumption from the sucrose. Subjects consuming the sweetener did not exhibit increases in energy intake, weight, and blood pressure that seen in the sucrose-consuming subjects [45]. Research in the metabolism of fructose has left more questions about the difference between short-term positive effects, and the negative effects of chronic, long-term use of fructose sugars [46]. The long-term negative effects can include changes in digestion, absorption, plasma hormone levels, appetite, and hepatic metabolism, leading to development of insulin resistance, diabetes, obesity, and inevitably cardiovascular disease.

When the metabolic pathways and characteristics of fructose are examined more closely, many of the questions about its positive and negative effects can be answered. Fructose is a potent regulator of glycogen synthesis and liver glucose uptake. Therefore any catalytic improvements are due to hepatic glucokinase and glucose uptake facilitation. However, as mentioned, the beneficial effects do not continue with chronic fructose utilization [47]. Because of its lipogenic properties, excess fructose in the diet can cause glucose and fructose malabsorption, and greater elevations in TG and cholesterol compared to other carbohydrates [48]. There are key differences in the metabolic pathways that glucose and fructose follow. Upon gastric absorption both fructose and glucose are delivered *via *the portal vein to the liver. It is believed that the ability of the liver to metabolize high doses of fructose is responsible for the disruption in energy stores and fuel metabolism observed [49-52]. In the liver, fructose is metabolized into glyceraldehyde and dihydroxyacetone phosphate. These particular fructose end products can then readily converge with the glycolytic pathway. Of key importance is the ability of fructose to by-pass the main regulatory step of glycolysis, the conversion of glucose-6-phosphate to fructose 1,6-bisphosphate, controlled by phosphofructokinase. Thus, while glucose metabolism is *negatively *regulated by phosphofructokinase, fructose can *continuously *enter the glycolytic pathway. Therefore, fructose can uncontrollably produce glucose, glycogen, lactate, and pyruvate, providing both the glycerol and acyl portions of acyl-glycerol molecules. These particular substrates, and the resultant excess energy flux due to unregulated fructose metabolism, will promote the over-production of TG (reviewed in [53]).

The glycemic index (GI) has been commonly used to differentiate and compare various nutrients, as well as to describe how different foods produce different plasma glucose levels after ingestion. The GI can range from 100 for glucose and baked potato compared to approximately 20 for fructose and whole barley [54]. Foods with varying GIs have different time courses associated with satiety. High GI carbohydrates have been reported to reduce appetite in the short term, whereas low GI carbohydrates possess a more delayed effect on energy intake controls [55]. Fructose appears to have differing effects on appetite compared to glucose, contributing to its negative properties. Anderson *et al*. determined the association between food intake and blood glucose, comparing glucose and a fructose mixture. Glucose was administered as a high GI preload, which resulted in lower mealtime energy intakes compared to the low GI preload of the glucose-fructose mixture. An inverse relationship was seen between GI (and blood glucose concentrations), and appetite with consequent increased food intakes seen with fructose [56]. In 2002, Vozzo *et al*. studied the comparative effects of glucose and fructose on blood glucose, insulin, and acute food intake. When subjects drank equienergetic preloads of glucose or fructose before an *ad libidum *buffet lunch, glucose concentrations were lower in the fructose group compared to glucose, and insulin concentrations were 50% higher in the fructose group in type 2 diabetics than in non-diabetics. The authors concluded that fructose may be a suitable replacement for glucose in diabetic patients – although it was found that satiating efficiencies of fructose certainly offered no advantages [57]. This study differs from others with regards to insulin secretion, but the trend is clear between GI, glucose concentrations, and appetite. An explanation for the variation in glucose and fructose glycemic responses appears to be dependent on rates of hydrolysis and absorption of glucose, and gastric emptying [58]. The variations observed in GI and appetite control of glucose and fructose can also be explained by differences in stimulation of insulin and leptin, important players in the long-term regulation of energy homeostasis. Fructose will generally produce smaller insulin excursions upon consumption because it does not stimulate the secretion of insulin from pancreatic beta cells, whereas glucose does. Insulin-regulated leptin will also have a reduced concentration and a decreased net effect on reducing appetite. Limited effects on appetite suppression, combined with the fact that fructose is favoured by the liver to be metabolized into lipid, will subsequently lead to weight gain, hyperinsulinemia, and the associated insulin resistance [59]. Glucose and fructose comparison studies continued examining new hormonal targets. In 2004, Teff *et al*. showed that subjects served meals with either 30% glucose beverages, or 30% fructose beverages, had differing hormonal and metabolic responses. Glycemic *excursions *and insulin responses were reduced by 66% and 65%, respectively, in the fructose-consuming subjects. There was a concomitant reduction in circulating leptin both in the short and long-term as well as a 30% reduction in ghrelin (an orexigenic gastroenteric hormone) in the fructose group compared to the glucose group. A prolonged elevation of TG was also seen in the high fructose subjects [60]. Both fat and fructose consumption usually results in low leptin concentrations which, in turn, leads to overeating in populations consuming energy from these particular macronutrients. An adipocyte hormone, adiponectin, also plays an important role in lipid homeostasis and insulin action [61]. The insulin sensitizer agonist, peroxisome proliferator-activated receptor-gamma, stimulates adiponectin production and adiponectin is in fact thought to be part of this agonist's mechanism lowering circulating fatty acids and increasing fat oxidation. The net effect is to decrease liver TG and increase insulin sensitivity [62]. Chronic fructose consumption reduces adiponectin responses, contributing to insulin resistance [63].

Animal studies have illustrated various differences between glucose and fructose metabolism. In 2002, Miller *et al*. injected fructose into the cerebroventricles of rats, and observed enhanced food intake, whereas similar concentrations of injected glucose suppressed appetite-agonist stimulated food intake [64]. Feeding rats either 32% glucose, fructose, or sucrose solutions, resulted in increased weight gain, and energy consumption compared to chow fed controls. Rats given the fructose and sucrose solutions also had a decreased ability to tolerate a glucose load, and fructose animals had greater serum TG levels over all other conditions ([65]. This is likely because the hepatic metabolism of fructose favours *de novo *lipogenesis. In combination with alterations in insulin signaling and leptin regulation, weight gain and unregulated energy intake can occur [33]. In 1986, Levine *et al*. found that fructose, administered in the form of the disaccharide sucrose, promotes obesity more than glucose because fructose does not stimulate thermogenesis [58]. These hormonal and physiological changes illustrate the important connections between energy intake, appetite control, weight gain, and insulin resistance.

## Fructose and insulin resistance

Increasingly, questions have been raised as to whether dietary carbohydrate and fructose intake are directly related to the development of type 2 diabetes. As insulin resistance is often associated with circulating C-peptide concentrations, a cross-sectional study was performed to assess dietary fructose and carbohydrate, and glycemic loads related to C-peptide concentrations. It was found that the highest quintile of fructose intake had 13.9% higher C-peptide concentrations than the lowest quintile. Of note, subjects with high intakes of cereal fiber had 15.6% lower C-peptide concentrations, indicating that these types of nutrients may have opposing roles in the development of insulin resistance [66]. A definite relationship has also been found between metabolic syndrome and hyperhomocysteinemia, which is associated with cardiovascular and cerebrovascular diseases. Rats fed a fructose-enriched diet had a 72% higher homocysteine levels after 5 weeks compared to chow-fed controls [67]. Elevated homocysteine levels are an important risk factor for vascular disease. Homocysteine was found to be higher in patients with stenotic vessels and coronary artery disease scores, and was in fact highest in diabetic patients [68]. This is consistent with the increased TG, very low density lipoprotein (VLDL) secretion, and atherosclerosis associated with chronic fructose feeding.

Although fructose does not appear to acutely increase insulin levels, chronic exposure seems to indirectly cause hyperinsulinemia and obesity through other mechanisms. One proposed mechanism involves GLUT5, a fructose transporter that is found to have significantly higher expression levels in young Zucker obese rats compared to lean controls. As the rats age and become diabetic, GLUT5 abundance and activity is compromised, causing an even more marked insulin resistance over lean rats, implying a possible role of GLUT5 receptors in the pathology of metabolic syndrome associated with fructose feeding and insulin resistance [69]. In rats fed 66% fructose for 2 weeks, insulin receptor mRNA, and subsequent insulin receptor numbers in skeletal muscle and liver were significantly lower compared to rats fed a standard chow diet. Also, blood pressure and plasma TG increased in the fructose-fed rats, even though there was no change in plasma insulin, glucose, or body weight [70]. Evidence shows these early steps in insulin signaling are important for insulin's metabolic effects. In a different study, it was found that after 28 days of fructose feeding there were no changes in insulin receptor concentration, but, insulin stimulated autophosphorylation, a mechanism necessary for insulin action, was reduced to 72% in the liver. Insulin receptor substrate (IRS) protein levels were similar, but there were significant decreases in insulin induced IRS (1/2) phosphorylation in both the liver and muscle of the fructose fed rats [71]. These changes are important, because it has been shown that the products of these insulin independent metabolic pathways lead to polyol formation and advanced glycation end products, which can contribute to the numerous complications and premature atherosclerosis seen in diabetic patients [58]. It is also known that such inflammations can lead to the pathogenesis of diabetes, and there is strong evidence suggesting that increased free fatty acids (FFA) in diabetic subjects and fructose fed models play a role in the inflammatory state of insulin resistance. If FFA are not removed from tissues, as occurs in fructose fed insulin resistant models, there is an increased energy and FFA flux that leads to the increased secretion of TG. Insulin resistance has also been correlated with intracellular TG stores, which are involved in lipotoxicity and beta cell failure leading to diabetes [72]. Another theory explaining how chronic fructose overnutrition can lead to type 2 diabetes is the hexosamine hypothesis, where hexosamine flux is thought to regulate glucose and satiety-sensing pathways. With overexpression of glutamine:fructose-6-phosphate amidotransferase, the key regulatory enzyme in hexosamine synthesis, the liver produces excess fatty acids, skeletal muscle becomes insulin resistant, and hyperinsulinemia results. This pathway of excess hexosamine flux leads to long-term storage of energy, and eventually obesity and type 2 diabetes [73].

## Fructose: a highly lipogenic nutrient

There is considerable evidence supporting the ability of high fructose diets to upregulate the lipogenesis pathway, leading to increased TG production [74]. Insulin and glucose are known to directly regulate lipid synthesis and secretion. Insulin controls hepatic sterol regulatory element binding protein (SREBP) expression, which is a key transcription factor responsible for regulating fatty acid and cholesterol biosynthesis. SREBP binds to sterol responsive elements (SRE) found on multiple genes, and can activate a cascade of enzymes involved in cholesterol biosynthetic pathways, such as HMG-CoA reductase [75] and fatty acid synthase (FAS) [76]. Miyazaki *et al*. reported an induction of the hepatic SREBP-1 isoform and lipogenic gene expression including FAS, acetyl-CoA carboxylase (ACC), and stearoyl-CoA desaturase (SCD) in mice following 7 days on a 60% fructose diet [77]. It is known that SREBPs are regulated by intracellular sterol concentrations. However, more recently, it has been established that hormones such as insulin and platelet derived growth factor play a role in regulating these transcription factors. Expression of SREBP is enhanced by insulin in all three major insulin target tissues, liver, fat, and skeletal muscle [78-81]. Similarly, levels of SREBP are enhanced in the presence of hyperinsulinemia [82,83]. There is evidence that the insulin-mediated stimulation of SREBP occurs through the MAP kinase pathway [84], with ERK1/2 being shown to activate the SREBP-1a isoform by phosphorylating serine 117) [85]. Despite the fact that SREBP-1 is directly stimulated via insulin signaling, the depletion of insulin and insulin signaling through streptozotocin (STZ) treatment paradoxically induces SREBP-1c expression upon glucose, fructose, or sucrose feeding. It would have been expected that SREBP-1c would be downregulated concomitantly along with the reduced insulin availability, but this is not the case. Glucose feeding causes a short-term peak induction, whereas fructose caused a gradual extended increase in SREBP-1c activity, providing evidence that lipogenesis can be independent of insulin signaling, given carbohydrate, and particularly fructose, availability [86].

Emerging evidence suggests that a protein phosphatase, known as PTP-1B, may link high carbohydrate feeding, insulin resistance, and lipogenesis. Recently, PTP-1B has been linked to lipogenesis and SREBP regulation. Shimizu *et al*. found that overexpression of protein tyrosine phosphatase 1B (PTP-1B), which is associated with dysfunctional insulin signaling, leads to increased mRNA and promoter activity of SREBP-1c, and subsequent increases in the expression of FAS. PTP-1B may therefore regulate the lipogenesis and hypertriglyceridemia associated with insulin resistance syndrome [87]. In insulin resistant fructose fed rats, it has been reported that the increase of hepatic SREBP-1 mRNA [88] occurs in correlation with an increased PTP-1B expression [87]. The authors established a role for PTP-1B in enhancing SREBP-1 gene expression through upregulation of Sp1 transcriptional activity, *via *an increase in protein phosphatase 2A activity [87]. FAS, an important downstream component of lipid synthesis, was extensively studied in rat livers. Dietary carbohydrates increased the transcriptional rate of FAS in comparison to proteins. Specifically, fructose feeding increased FAS mRNA concentrations, and somewhat increased transcriptional rate. This suggests that fructose may increase the stability of FAS mRNA, while carbohydrates stimulate FAS through increased transcriptional rate [89]. Other studies using animal models of insulin resistance, for example, the Wistar fatty rats, showed the effects of dietary carbohydrates on TG production. Feeding rats fructose stimulated FAS, and created a 56% increase in TG secretion rate, and an 86% increase in plasma TG. Feeding glucose, however, did not have this effect on TG production, nor did it affect induction of FAS. This is likely because glucose stimulates both TG production, and TG removal, maintaining homeostasis. Fructose stimulates TG production, but impairs removal, creating the known dyslipidemic profile [90]. The human liver possesses a large capacity to metabolize fructose to lipids because of its ability to shunt metabolism toward serum TG production. TG stores supply an energy 'sink', providing an almost unlimited TG production capacity. Conversely, glucose as opposed to fructose would decrease serum TG [91]. As discussed earlier, the effects of fructose in promoting TG synthesis are independent of insulinemia. Hirsch argued that carbohydrate overload results in elevated TG because the large amounts of sugar that need to be absorbed so rapidly from the intestine lead to the involvement of other metabolic pathways, such as the hexose monophosphate shunt, that that favour the synthesis of FFA [92]. Again, the liver takes up dietary fructose rapidly where it can be converted to glycerol-3-phosphate. This substrate favours esterification of unbound FFA to form the TG [93]. It has also been found that increases of 1,2-sn-diacylglycerol and elevated expression of a PKC isoenzyme are associated with the enhanced synthesis of TG observed with high fructose diet models [94]. In these scenarios, where there is excess hepatic fatty acid uptake, synthesis and secretion, 'input' of fats in the liver exceed 'outputs', and hepatic steatosis occurs [95]. The mechanisms of steatosis and liver enlargement due to fructose intake are not well understood, but it is believed to be related to microsomal enzyme induction, increased storage of lipids, peroxisome proliferation, and hyperfunction due to excessive hepatic 'workloads' [96]. All of these factors contribute to fructose being a highly lipogenic nutrient, and to the resultant hepatic steatosis.

## Mechanisms of fructose induced lipoprotein overproduction

**Figure 1:**
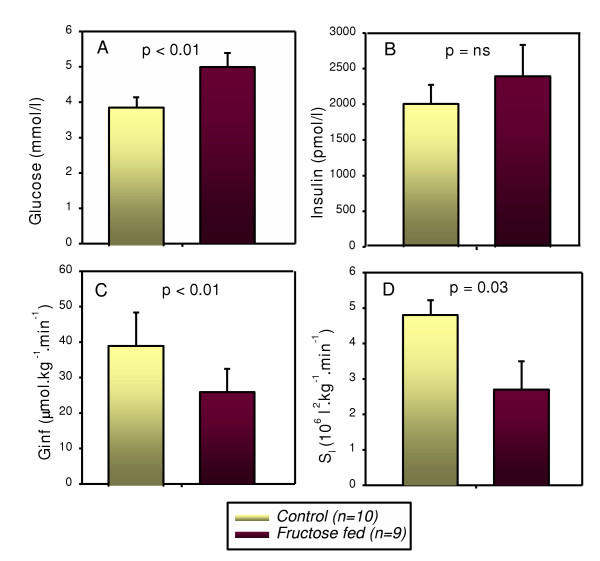
**Fructose-induced insulin resistance: evidence from euglycemic hyperinsulinemic clamp studies. **Mean glucose levels (**A**) were slightly but significantly higher in fructose-fed vs. control animals during the last 30 mins of the clamp period (p < 0.01). Mean insulin levels (**B**) were slightly but not significantly higher in the fructose-fed vs. control hamsters during the clamp period. The glucose infusion rate (Ginf) (**C**) during the clamp period was significantly lower in fructose-fed vs. control animals (p < 0.01). The calculated insulin sensitivity index (S_I _– see methods) (**D**) was also significantly lower in the fructose-fed vs. control hamsters (p = 0.03). Fructose-fed (n = 9), control hamsters (n = 10). (*adapted from Taghibiglou et al. [100]*).

There is growing evidence that the insulin resistant state developed upon fructose feeding is also associated with stimulated hepatic VLDL secretion. Several animal models have been employed to examine the mechanisms of this induction of VLDL, and the subsequent increases in plasma TG observed. Mechanistic studies based on carbohydrate versus lipid metabolism have recently become important because carbohydrate induced hypertriglyceridemia shares a metabolic basis with high fat diet induced endogenous hypertriglycerolemia. The similarly induced dyslipidemias would therefore have the same or similar atherogenic risks [97]. Carbohydrate induced hypertriglycerolemia results from a combination of both TG overproduction, and inadequate TG clearance [97,98]. These disease processes and the hepatic steatosis caused by stimulated lipogenesis have been illustrated by fructose fed animal models showing how aberrant leptin signaling, hyperinsulinemia, and dyslipidemia are related to TG induction [95]. Animals maintained on a chronic high fructose diet develop elevated NEFA and hyperinsulinemia at the expense of glycemic control [99]. This is not surprising, as fructose-induced metabolic dyslipidemia is usually accompanied by whole body insulin resistance [100] and reduced hepatic insulin sensitivity [101]. In the fructose fed hamster model, animals showed decreased glucose disappearance rates, increased plasma NEFA and increased plasma and liver TG [27]. Figure 1 (adapted from ref. 100) shows clear *in vivo *evidence of fructose-induced insulin resistance as assessed by euglycemic hyperinsulinemic clamp studies. Taghibiglou *et al*. further characterized the fructose fed hamster model demonstrating the development of a metabolic dyslipidemic state characterized by high plasma levels of VLDL-TG and apolipoprotein B (apoB) due to hepatic lipoprotein overproduction [100]. Serum TGs are elevated *via *both an increased secretion, and decreased clearance of VLDL [102]. Also, high rates of lipolysis in visceral adipose depots can increase availability of NEFAs and promote hepatic TG synthesis. The TG is then packaged with apoB, and secreted as VLDL particles [93]. Evidence has shown that there is a complex interplay of cellular enzymes regulating lipid synthesis and uptake, as well as export and oxidation. Observations of the actions of insulin affecting lipid secretion as well as inhibition of TG has brought research interests towards the effects of chronic insulin stimulation on VLDL secretion and transport. Excess VLDL secretion has been shown to deliver increased fatty acids and TG to muscle and other tissues, further inducing insulin resistance [103]. Induced cellular changes include alterations in hepatic pyruvate dehydrogenase, changes in insulin signaling phosphorylation, and increases of inflammatory cytokines [104,105]. It is evident that the metabolic effects of fructose occur through rapid utilization in the liver due to the bypassing of the regulatory phosphofructokinase step in glycolysis. This in turn causes activation of pyruvate dehydrogenase, and subsequent modifications favoring esterification of fatty acids, again leading to increased VLDL secretion [53]. Increases in VLDL secretion can then lead to chain reactions in other lipoproteins and lipids, such as low density lipoprotein (LDL). Resultant LDL cholesterol levels induced by high fructose intake are illustrated by comparison of a diet including 20% fructose, contrasted to a starch diet of less than 3% fructose. The 20% fructose diet initiated a cycle of increased fasting serum total and LDL cholesterol of 9% and 11%, respectively, over the starch feeding [106]. Increased evidence was shown in transgenic apo AI-CIII-AIV mice, fed a fructose solution for 9 months, where differential expressions of the apo AI and apo AIV genes were found. This indicated general perturbations in response to dietary intakes, causing long-term adverse effects in this hyperlipidemia mouse model [107]. The male Wistar fatty rat model of obese type 2 diabetes has also shown hyperglycemia. Remarkably, the female Wistar rats only develop this hyperglycemia when given sucrose, containing the responsible element of fructose, which causes increases in gluconeogenic enzymes and decreases in glucokinase. A hypertriglyceridemic effect is seen, presumably due to hepatic overproductions of VLDL and induction of lipogenic enzymes *via *dietary fructose [108].

Another contributing factor to VLDL overproduction includes fructose effects on lipid peroxidation. High fructose diets can have a hypertriglyceridemic and pro-oxidant effect, and fructose fed rats have shown less protection from lipid peroxidation. Replacing the fructose in these diets with a more natural source of high fructose, honey, reduces this susceptibility and lowers plasma nitrite and nitrate levels [109]. In 2004, Kelley *et al*. hypothesized that pro-oxidant stress response pathways may mediate hepatic increases in VLDL secretion and delayed clearance upon fructose feeding. Hypertriglyceridemic fructose fed rats were treated with lipoxygenase inhibitors, which reversed the inflammatory protein activity response, and the lipid dysregulation observed [102]. Recent findings have also shown that the hyperlipidemic and pro-oxidant effect induced by a high fructose diet can be decreased by oligofructose consumption. Oligofructose administered to fructose fed rats did not alter insulin concentrations, and lowered plasma leptin by 50% compared to control groups. Oligofructose prevented TG changes induced by fructose feeding, and decreased hepatic TG accumulation. The peroxidation effect of fructose was also decreased by oligofructose, and had beneficial protective effects [110]. Oxidative stress has often been implicated in the pathology of insulin resistance induced by fructose feeding, and lipid peroxides, diene conjugates, and reactive substances are undeniably elevated in fructose fed animals, especially accompanying a deficient antioxidant system. Administration of alpha-lipoic acid (LA) has been shown to prevent these changes, and improve insulin sensitivity [111]. LA treatment also prevents several deleterious effects of fructose feeding: the increases in cholesterol, TG, activity of lipogenic enzymes, and VLDL secretion, the reductions in lipoprotein lipase and HDL cholesterol and may even normalize a dyslipidemic cholesterol distribution of plasma lipoproteins [112]. Taken together, this evidence shows a clear role of peroxidative stress pathways involved in VLDL oversecretion.

Observations made in our own laboratory have also shown aberrant lipogenesis activity. In primary hepatocytes isolated from fructose fed hamsters, there were significant increases in LXRα, SREBP-1, FAS and SCD, which indicate increased activity of the lipogenic pathways (unpublished observations). Fructose has also been implicated in reducing PPARα levels in rat hepatocytes. PPARα is a ligand activated nuclear hormone receptor that is responsible for inducing mitochondrial and peroxisomal β-oxidation. Nagai *et al*. found that following 8 weeks of a high fructose diet, rats showed decreased PPARα mRNA and protein levels [88]. In addition, primary rat hepatocytes treated with fructose also showed decreased PPARα expression, suggesting that fructose or its metabolites can directly regulate lipid oxidation. We have also recently detected decreased mRNA levels of PPARα in both liver and intestine of the fructose fed hamster (unpublished observations). Hence, decreased PPARα expression can result in reduced oxidation, leading to cellular lipid accumulation. For example, PPARα null mice have extensive hepatic steatosis because of diminished β-oxidation capacity, such as seen in the insulin resistant state [113]. Other mechanisms have been illustrated by Taghibiglou *et al*., who found evidence for enhanced lipoprotein assembly, reduced intracellular apoB degradation, and increased microsomal triglyceride transfer protein (MTP) mass, mRNA and activity in the fructose fed hamster [100]. These metabolic changes also coincided with a decrease in ER-60, a cysteine protease that may play a role in apoB degradation, and an increase in synthesis and secretion of apoB [101]. It appears that a complex relationship exists in the fructose fed animal model that links insulin resistance and dyslipidemia through NEFA flux, SREBP-1 expression, *de novo *lipogenesis and MTP expression. Amplified MTP activity and expression would be expected to stimulate the assembly and secretion of apoB-lipoproteins, as an association has been demonstrated between MTP levels and VLDL production [114]. As insulin is a negative regulator of MTP gene expression [115], the upregulation of MTP that has been observed in insulin resistance states is predictable. MTP is also negatively regulated by SREBP through sterol response element (SRE) regions located within -124 and -116 of the 5' MTP gene promoter [116]. However, in fructose fed animals [87] as well as other models of insulin resistance [117] where increased levels of MTP and SREBP have been established, the regulatory effects of SREBP may play a minor role in regulating MTP expression. Rather increased hepatic NEFA and increased TG stores might stimulate MTP expression [118]. Recent observations in our laboratory show that oleic acid can stimulate the MTP promoter and the stimulation occurs independently of SRE activity (unpublished observations). Thus, in insulin resistance states, increased MTP may occur through another mechanism that may block SREBP-mediated inhibition of the promoter. These phenomena help explain the increased assembly and secretion of apoB in fructose fed models. In addition, increased levels of small dense LDL particles have been observed in insulin resistant states [119]. Early studies by Verschoor *et al*. showed that fructose diets altered the structure and function of VLDL particles causing and increase in the TG: protein ratio, and an increased total cholesterol and phospholipid content [120]. LDL particle size has been found to be inversely related to TG concentration [121] and therefore the higher TG results in a smaller, denser, more atherogenic LDL particle, which contributes to the morbidity of the metabolic disorders associated with insulin resistance. Several theories are proposed for the overproduction of VLDL: more TG per VLDL particle, increases in particle number, changes in the production rates of VLDL TG or apoB, decreased TG clearance, increased lipoprotein lipase activity, and increased *de novo *lipogenesis. It is likely a combination of some or all of these factors that contribute to the elevated TG seen in a fructose rich carbohydrate fed model of metabolic disorder. High fructose, which stimulates VLDL secretion, may initiate the cycle that results in metabolic syndrome long before type 2 diabetes and obesity develop [103].

More recently, our studies have identified an interesting link between the development of insulin resistance and deregulation of intestinal lipoprotein metabolism [122]. Chronic fructose feeding stimulated intestinal secretion of apolipoprotein B48-containing lipoprotein particles accompanied by enhanced intestinal lipid synthesis in the form of free cholesterol, cholesterol ester, and triglyceride, as well as increases in both MTP mass and activity. These results suggest that in insulin resistant or diabetic animals, there may be a mechanism causing enhanced intestinal secretion of lipoproteins in the fasting state. Fructose feeding may enhance this basal level of lipoprotein secretion through increased *de novo *lipogenesis and increased MTP availability. Comparison of plasma lipoproteins from fructose-fed animals showed a significant shift toward secretion of larger, less dense, chylomicrons in the insulin resistant animals [123].

## Concluding remarks

**Figure 2:**
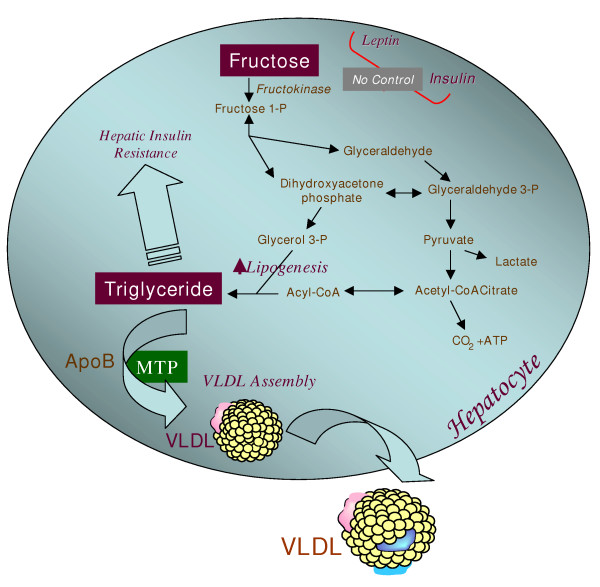
**Hepatic fructose metabolism: A highly lipogenic pathway. **Fructose is readily absorbed from the diet and rapidly metabolized principally in the liver. Fructose can provide carbon atoms for both the glycerol and the acyl portions of triglyceride. Fructose is thus a highly efficient inducer of *de novo *lipogenesis. High concentrations of fructose can serve as a relatively unregulated source of acetyl CoA. In contrast to glucose, dietary fructose does NOT stimulate insulin or leptin (which are both important regulators of energy intake and body adiposity). Stimulated triglyceride synthesis is likely to lead to hepatic accumulation of triglyceride, which has been shown to reduce hepatic insulin sensitivity, as well as increased formation of VLDL particles due to higher substrate availability, increased apoB stability, and higher MTP, the critical factor in VLDL assembly.

The alarming increase in fructose consumption may be an important contributor to the epidemic of obesity and insulin resistant diabetes in both pediatric and adult populations. For thousands of years, the human diet contained a relatively small amount of naturally occurring fructose from fruits and other complex foods. Adaptation of humans to a high glucose/low fructose diet has meant that hepatic carbohydrate metabolism is designed to actively metabolize glucose with a limited capacity for metabolizing a small daily intake of fructose. The increasing application of high fructose sweeteners over the past few decades has resulted in a considerable rise in the dietary intake of fructose. A high flux of fructose to the liver, the main organ capable of metabolizing this simple carbohydrate, disturbs normal hepatic carbohydrate metabolism leading to two major consequences (Figure 2): perturbations in glucose metabolism and glucose uptake pathways, and a significantly enhanced rate of *de novo *lipogenesis and TG synthesis, driven by the high flux of glycerol and acyl portions of TG molecules coming from fructose catabolism. These metabolic disturbances appear to underlie the induction of insulin resistance commonly observed with high fructose feeding in both humans and animal models. Fructose induced insulin resistant states are commonly characterized by a profound metabolic dyslipidemia, which appears to result from hepatic and intestinal overproduction of atherogenic lipoprotein particles. Taking into consideration that a typical western diet not only contains high levels of fructose but is also rich in both fat and cholesterol, synergistic interactions among these nutrients can readily occur leading to a greater degree of insulin resistance and dyslipidemia. In conclusion, emerging evidence from recent epidemiological and biochemical studies clearly suggests that the high dietary intake of fructose has rapidly become an important causative factor in the development of the metabolic syndrome. There is an urgent need for increased public awareness of the risks associated with high fructose consumption and greater efforts should be made to curb the supplementation of packaged foods with high fructose additives.

## List of Abbreviations

ACC: acetyl-CoA carboxylase

apoB: apolipoprotein B

ERK: extracellular signal related kinase

FAS: fatty acid synthase

GSK-3: glycogen synthase kinase-3

HDL: high density lipoprotein

HFCS: high fructose corn syrup

IR: insulin receptor

IRS: insulin receptor substrate

LA: alpha-lipoic acid

LDL: low density lipoprotein

LXR: liver X receptor

MAPK: mitogen activated protein kinase

MTP: microsomal triglyceride transfer protein

NEFA: non-esterified fatty acids

PA-1: plasminogen activator inhibitor-1

PI3-kinase: phosphatidylinositol 3 kinase

PKB: protein kinase B

PKC: protein kinase C

PPAR: peroxisome proliferator activated receptor

PTP-1B: protein tyrosine phosphatase-1B

SCD: stearoyl-CoA desaturase

SREBP: sterol regulatory element binding protein

TG: triglyceride

VLDL: very low density lipoprotein

## References

[B1] Astrup A, Finer N (2000). Redefining type 2 diabetes: 'diabesity' or 'obesity dependent diabetes mellitus'?. Obes Rev.

[B2] Mokdad AH, Ford ES, Bowman BA, Nelson DE, Engelgau MM, Vinicor F, Marks JS (2000). Diabetes trends in the US: 1990–1998. Diabetes Care.

[B3] Mokdad AH, Bowman BA, Ford ES, Vinicor F, Marks JS, Koplan JP (2001). The continuing epidemics of obesity and diabetes in the United States. Jama.

[B4] Mokdad AH, Serdula MK, Dietz WH, Bowman BA, Marks JS, Koplan JP (1999). The spread of the obesity epidemic in the United States, 1991–1998. Jama.

[B5] Pan XR, Yang WY, Li GW, Liu J (1997). Prevalence of diabetes and its risk factors in China, 1994. National Diabetes Prevention and Control Cooperative Group. Diabetes Care.

[B6] Ramachandran A, Snehalatha C, Latha E, Vijay V, Viswanathan M (1997). Rising prevalence of NIDDM in an urban population in India. Diabetologia.

[B7] Centers for Disease Control and Prevention NCfHS, Division of Health Interview Statistics (1997). Census of the population and population estimates.

[B8] Zimmet P, Alberti KG, Shaw J (2001). Global and societal implications of the diabetes epidemic. Nature.

[B9] Keller KB, Lemberg L (2003). Obesity and the metabolic syndrome. Am J Crit Care.

[B10] Songer TJ (1992). The economic costs of NIDDM. Diabetes Metab Rev.

[B11] (2001). Executive Summary of The Third Report of The National Cholesterol Education Program (NCEP) Expert Panel on Detection, Evaluation, And Treatment of High Blood Cholesterol In Adults (Adult Treatment Panel III). Jama.

[B12] Edwards KL, Talmud PJ, Newman B, Krauss RM, Austin MA (2001). Lipoprotein candidate genes for multivariate factors of the insulin resistance syndrome: a sib-pair linkage analysis in women twins. Twin Res.

[B13] Avramoglu RK, Qiu W, Adeli K (2003). Mechanisms of metabolic dyslipidemia in insulin resistant states: deregulation of hepatic and intestinal lipoprotein secretion. Front Biosci.

[B14] Rosmond R (2005). Role of stress in the pathogenesis of the metabolic syndrome. Psychoneuroendocrinology.

[B15] Bao W, Srinivasan SR, Berenson GS (1996). Persistent elevation of plasma insulin levels is associated with increased cardiovascular risk in children and young adults. The Bogalusa Heart Study. Circulation.

[B16] Freedman DS, Khan LK, Dietz WH, Srinivasan SR, Berenson GS (2001). Relationship of childhood obesity to coronary heart disease risk factors in adulthood: the Bogalusa Heart Study. Pediatrics.

[B17] Valek J, Vlasakova Z (1997). [The metabolic syndrome, its heredity, methods of detection and clinical significance]. Vnitr Lek.

[B18] Kohen-Avramoglu R, Theriault A, Adeli K (2003). Emergence of the metabolic syndrome in childhood: an epidemiological overview and mechanistic link to dyslipidemia. Clin Biochem.

[B19] Feskens EJ, Virtanen SM, Rasanen L, Tuomilehto J, Stengard J, Pekkanen J, Nissinen A, Kromhout D (1995). Dietary factors determining diabetes and impaired glucose tolerance. A 20-year follow-up of the Finnish and Dutch cohorts of the Seven Countries Study. Diabetes Care.

[B20] Hill JO, Lin D, Yakubu F, Peters JC (1992). Development of dietary obesity in rats: influence of amount and composition of dietary fat. Int J Obes Relat Metab Disord.

[B21] Kromhout D, Menotti A, Bloemberg B, Aravanis C, Blackburn H, Buzina R, Dontas AS, Fidanza F, Giampaoli S, Jansen A (1995). Dietary saturated and trans fatty acids and cholesterol and 25-year mortality from coronary heart disease: the Seven Countries Study. Prev Med.

[B22] Romieu I, Willett WC, Stampfer MJ, Colditz GA, Sampson L, Rosner B, Hennekens CH, Speizer FE (1988). Energy intake and other determinants of relative weight. Am J Clin Nutr.

[B23] Liu S, Manson JE (2001). Dietary carbohydrates, physical inactivity, obesity, and the 'metabolic syndrome' as predictors of coronary heart disease. Curr Opin Lipidol.

[B24] Jenkins DJ, Wolever TM, Taylor RH, Barker H, Fielden H, Baldwin JM, Bowling AC, Newman HC, Jenkins AL, Goff DV (1981). Glycemic index of foods: a physiological basis for carbohydrate exchange. Am J Clin Nutr.

[B25] Jenkins DJ, Jenkins AL (1987). The glycemic index, fiber, and the dietary treatment of hypertriglyceridemia and diabetes. J Am Coll Nutr.

[B26] Miller JC (1994). Importance of glycemic index in diabetes. Am J Clin Nutr.

[B27] Kasim-Karakas SE, Vriend H, Almario R, Chow LC, Goodman MN (1996). Effects of dietary carbohydrates on glucose and lipid metabolism in golden Syrian hamsters. J Lab Clin Med.

[B28] Hwang IS, Ho H, Hoffman BB, Reaven GM (1987). Fructose-induced insulin resistance and hypertension in rats. Hypertension.

[B29] Cummings LE (1988). Commercial foodservice considerations in providing consumer-driven nutrition program elements. Part I. Consumer health objectives and associated employee education needs. Cater Health.

[B30] Willett WC (2002). Dietary fat plays a major role in obesity: no. Obes Rev.

[B31] Kromhout D (2001). Diet and cardiovascular diseases. J Nutr Health Aging.

[B32] Gross LS, Li L, Ford ES, Liu S (2004). Increased consumption of refined carbohydrates and the epidemic of type 2 diabetes in the United States: an ecologic assessment. Am J Clin Nutr.

[B33] Bray GA, Nielsen SJ, Popkin BM (2004). Consumption of high-fructose corn syrup in beverages may play a role in the epidemic of obesity. Am J Clin Nutr.

[B34] Putnam J (1999). Food consumption, prices, and expenditures, 1970–91. Economic Research Service.

[B35] United States Dept of Agriculture (1992). The Food guide pyramid.

[B36] Cavadini C, Siega-Riz AM, Popkin BM (2000). US adolescent food intake trends from 1965 to 1996. West J Med.

[B37] Jacobson MF (2004). High-fructose corn syrup and the obesity epidemic. Am J Clin Nutr.

[B38] Bowman SA (2002). Beverage choices of young females: changes and impact on nutrient intakes. J Am Diet Assoc.

[B39] Wharton CM, Hampl JS (2004). Beverage consumption and risk of obesity among Native Americans in Arizona. Nutr Rev.

[B40] Mehnert H (1976). [Sugar substitutes in the diabetic diet]. Int Z Vitam Ernahrungsforsch Beih.

[B41] Moore MC, Cherrington AD, Mann SL, Davis SN (2000). Acute fructose administration decreases the glycemic response to an oral glucose tolerance test in normal adults. J Clin Endocrinol Metab.

[B42] (2004). Position of the American Dietetic Association: use of nutritive and nonnutritive sweeteners. J Am Diet Assoc.

[B43] Hung CT (1989). Effects of high-fructose (90%) corn syrup on plasma glucose, insulin, and C-peptide in non-insulin-dependent diabetes mellitus and normal subjects. Taiwan Yi Xue Hui Za Zhi.

[B44] Breinholt VM, Nielsen SE, Knuthsen P, Lauridsen ST, Daneshvar B, Sorensen A (2003). Effects of commonly consumed fruit juices and carbohydrates on redox status and anticancer biomarkers in female rats. Nutr Cancer.

[B45] Raben A, Vasilaras TH, Moller AC, Astrup A (2002). Sucrose compared with artificial sweeteners: different effects on ad libitum food intake and body weight after 10 wk of supplementation in overweight subjects. Am J Clin Nutr.

[B46] Moyer AE, Rodin J (1993). Fructose and behavior: does fructose influence food intake and macronutrient selection?. Am J Clin Nutr.

[B47] McGuinness OP, Cherrington AD (2003). Effects of fructose on hepatic glucose metabolism. Curr Opin Clin Nutr Metab Care.

[B48] Hallfrisch J (1990). Metabolic effects of dietary fructose. Faseb J.

[B49] Daly ME, Vale C, Walker M, Alberti KG, Mathers JC (1997). Dietary carbohydrates and insulin sensitivity: a review of the evidence and clinical implications. Am J Clin Nutr.

[B50] Commerford SR, Ferniza JB, Bizeau ME, Thresher JS, Willis WT, Pagliassotti MJ (2002). Diets enriched in sucrose or fat increase gluconeogenesis and G-6-Pase but not basal glucose production in rats. Am J Physiol Endocrinol Metab.

[B51] Dirlewanger M, Schneiter P, Jequier E, Tappy L (2000). Effects of fructose on hepatic glucose metabolism in humans. Am J Physiol Endocrinol Metab.

[B52] Pagliassotti MJ, Wei Y, Bizeau ME (2003). Glucose-6-phosphatase activity is not suppressed but the mRNA level is increased by a sucrose-enriched meal in rats. J Nutr.

[B53] Mayes PA (1993). Intermediary metabolism of fructose. Am J Clin Nutr.

[B54] Truswell AS (1992). Glycaemic index of foods. Eur J Clin Nutr.

[B55] Anderson GH, Woodend D (2003). Effect of glycemic carbohydrates on short-term satiety and food intake. Nutr Rev.

[B56] Anderson GH, Catherine NL, Woodend DM, Wolever TM (2002). Inverse association between the effect of carbohydrates on blood glucose and subsequent short-term food intake in young men. Am J Clin Nutr.

[B57] Vozzo R, Baker B, Wittert GA, Wishart JM, Morris H, Horowitz M, Chapman I (2002). Glycemic, hormone, and appetite responses to monosaccharide ingestion in patients with type 2 diabetes. Metabolism.

[B58] Levine R (1986). Monosaccharides in health and disease. Annu Rev Nutr.

[B59] Elliott SS, Keim NL, Stern JS, Teff K, Havel PJ (2002). Fructose, weight gain, and the insulin resistance syndrome. Am J Clin Nutr.

[B60] Teff KL, Elliott SS, Tschop M, Kieffer TJ, Rader D, Heiman M, Townsend RR, Keim NL, D'Alessio D, Havel PJ (2004). Dietary fructose reduces circulating insulin and leptin, attenuates postprandial suppression of ghrelin, and increases triglycerides in women. J Clin Endocrinol Metab.

[B61] Mora S, Pessin JE (2002). An adipocentric view of signaling and intracellular trafficking. Diabetes Metab Res Rev.

[B62] Yamauchi T, Kamon J, Minokoshi Y, Ito Y, Waki H, Uchida S, Yamashita S, Noda M, Kita S, Ueki K (2002). Adiponectin stimulates glucose utilization and fatty-acid oxidation by activating AMP-activated protein kinase. Nat Med.

[B63] Havel PJ (2002). Control of energy homeostasis and insulin action by adipocyte hormones: leptin, acylation stimulating protein, and adiponectin. Curr Opin Lipidol.

[B64] Miller CC, Martin RJ, Whitney ML, Edwards GL (2002). Intracerebroventricular injection of fructose stimulates feeding in rats. Nutr Neurosci.

[B65] Kanarek RB, Orthen-Gambill N (1982). Differential effects of sucrose, fructose and glucose on carbohydrate-induced obesity in rats. J Nutr.

[B66] Wu T, Giovannucci E, Pischon T, Hankinson SE, Ma J, Rifai N, Rimm EB (2004). Fructose, glycemic load, and quantity and quality of carbohydrate in relation to plasma C-peptide concentrations in US women. Am J Clin Nutr.

[B67] Oron-Herman M, Rosenthal T, Sela BA (2003). Hyperhomocysteinemia as a component of syndrome X. Metabolism.

[B68] Okada E, Oida K, Tada H, Asazuma K, Eguchi K, Tohda G, Kosaka S, Takahashi S, Miyamori I (1999). Hyperhomocysteinemia is a risk factor for coronary arteriosclerosis in Japanese patients with type 2 diabetes. Diabetes Care.

[B69] Litherland GJ, Hajduch E, Gould GW, Hundal HS (2004). Fructose transport and metabolism in adipose tissue of Zucker rats: diminished GLUT5 activity during obesity and insulin resistance. Mol Cell Biochem.

[B70] Catena C, Giacchetti G, Novello M, Colussi G, Cavarape A, Sechi LA (2003). Cellular mechanisms of insulin resistance in rats with fructose-induced hypertension. Am J Hypertens.

[B71] Ueno M, Bezerra RM, Silva MS, Tavares DQ, Carvalho CR, Saad MJ (2000). A high-fructose diet induces changes in pp185 phosphorylation in muscle and liver of rats. Braz J Med Biol Res.

[B72] Ziegler O, Quilliot D, Guerci B, Drouin P (2001). [Macronutrients, fat mass, fatty acid flux and insulin sensitivity]. Diabetes Metab.

[B73] McClain DA (2002). Hexosamines as mediators of nutrient sensing and regulation in diabetes. J Diabetes Complications.

[B74] Kok N, Roberfroid M, Delzenne N (1996). Dietary oligofructose modifies the impact of fructose on hepatic triacylglycerol metabolism. Metabolism.

[B75] Brown MS, Goldstein JL (1997). The SREBP pathway: regulation of cholesterol metabolism by proteolysis of a membrane-bound transcription factor. Cell.

[B76] Bennett MK, Lopez JM, Sanchez HB, Osborne TF (1995). Sterol regulation of fatty acid synthase promoter. Coordinate feedback regulation of two major lipid pathways. J Biol Chem.

[B77] Miyazaki M, Dobrzyn A, Man WC, Chu K, Sampath H, Kim HJ, Ntambi JM (2004). Stearoyl-CoA desaturase 1 gene expression is necessary for fructose-mediated induction of lipogenic gene expression by sterol regulatory element-binding protein-1c-dependent and -independent mechanisms. J Biol Chem.

[B78] Sewter C, Berger D, Considine RV, Medina G, Rochford J, Ciaraldi T, Henry R, Dohm L, Flier JS, O'Rahilly S, Vidal-Puig AJ (2002). Human obesity and type 2 diabetes are associated with alterations in SREBP1 isoform expression that are reproduced ex vivo by tumor necrosis factor-alpha. Diabetes.

[B79] Kim JB, Sarraf P, Wright M, Yao KM, Mueller E, Solanes G, Lowell BB, Spiegelman BM (1998). Nutritional and insulin regulation of fatty acid synthetase and leptin gene expression through ADD1/SREBP1. J Clin Invest.

[B80] Guillet-Deniau I, Mieulet V, Le Lay S, Achouri Y, Carre D, Girard J, Foufelle F, Ferre P (2002). Sterol regulatory element binding protein-1c expression and action in rat muscles: insulin-like effects on the control of glycolytic and lipogenic enzymes and UCP3 gene expression. Diabetes.

[B81] Foretz M, Guichard C, Ferre P, Foufelle F (1999). Sterol regulatory element binding protein-1c is a major mediator of insulin action on the hepatic expression of glucokinase and lipogenesis-related genes. Proc Natl Acad Sci U S A.

[B82] Boizard M, Le Liepvre X, Lemarchand P, Foufelle F, Ferre P, Dugail I (1998). Obesity-related overexpression of fatty-acid synthase gene in adipose tissue involves sterol regulatory element-binding protein transcription factors. J Biol Chem.

[B83] Shimomura I, Bashmakov Y, Horton JD (1999). Increased levels of nuclear SREBP-1c associated with fatty livers in two mouse models of diabetes mellitus. J Biol Chem.

[B84] Kotzka J, Lehr S, Roth G, Avci H, Knebel B, Muller-Wieland D (2004). Insulin-activated Erk-mitogen-activated protein kinases phosphorylate sterol regulatory element-binding Protein-2 at serine residues 432 and 455 in vivo. J Biol Chem.

[B85] Roth G, Kotzka J, Kremer L, Lehr S, Lohaus C, Meyer HE, Krone W, Muller-Wieland D (2000). MAP kinases Erk1/2 phosphorylate sterol regulatory element-binding protein (SREBP)-1a at serine 117 in vitro. J Biol Chem.

[B86] Matsuzaka T, Shimano H, Yahagi N, Amemiya-Kudo M, Okazaki H, Tamura Y, Iizuka Y, Ohashi K, Tomita S, Sekiya M (2004). Insulin-independent induction of sterol regulatory element-binding protein-1c expression in the livers of streptozotocin-treated mice. Diabetes.

[B87] Shimizu S, Ugi S, Maegawa H, Egawa K, Nishio Y, Yoshizaki T, Shi K, Nagai Y, Morino K, Nemoto K (2003). Protein-tyrosine phosphatase 1B as new activator for hepatic lipogenesis via sterol regulatory element-binding protein-1 gene expression. J Biol Chem.

[B88] Nagai Y, Nishio Y, Nakamura T, Maegawa H, Kikkawa R, Kashiwagi A (2002). Amelioration of high fructose-induced metabolic derangements by activation of PPARalpha. Am J Physiol Endocrinol Metab.

[B89] Katsurada A, Iritani N, Fukuda H, Matsumura Y, Nishimoto N, Noguchi T, Tanaka T (1990). Effects of nutrients and hormones on transcriptional and post-transcriptional regulation of fatty acid synthase in rat liver. Eur J Biochem.

[B90] Kazumi T, Odaka H, Hozumi T, Ishida Y, Amano N, Yoshino G (1997). Effects of dietary fructose or glucose on triglyceride production and lipogenic enzyme activities in the liver of Wistar fatty rats, an animal model of NIDDM. Endocr J.

[B91] Herman RH, Zakim D, Stifel FB (1970). Effect of diet on lipid metabolism in experimental animals and man. Fed Proc.

[B92] Hirsch J (1995). Role and benefits of carbohydrate in the diet: key issues for future dietary guidelines. Am J Clin Nutr.

[B93] Fried SK, Rao SP (2003). Sugars, hypertriglyceridemia, and cardiovascular disease. Am J Clin Nutr.

[B94] Donnelly R, Reed MJ, Azhar S, Reaven GM (1994). Expression of the major isoenzyme of protein kinase-C in skeletal muscle, nPKC theta, varies with muscle type and in response to fructose-induced insulin resistance. Endocrinology.

[B95] Koteish A, Diehl AM (2001). Animal models of steatosis. Semin Liver Dis.

[B96] Bar A (1999). Characteristics and significance of D-tagatose-induced liver enlargement in rats: An interpretative review. Regul Toxicol Pharmacol.

[B97] Parks EJ, Hellerstein MK (2000). Carbohydrate-induced hypertriacylglycerolemia: historical perspective and review of biological mechanisms. Am J Clin Nutr.

[B98] Kazumi T, Vranic M, Steiner G (1986). Triglyceride kinetics: effects of dietary glucose, sucrose, or fructose alone or with hyperinsulinemia. Am J Physiol.

[B99] Thorburn AW, Storlien LH, Jenkins AB, Khouri S, Kraegen EW (1989). Fructose-induced in vivo insulin resistance and elevated plasma triglyceride levels in rats. Am J Clin Nutr.

[B100] Taghibiglou C, Carpentier A, Van Iderstine SC, Chen B, Rudy D, Aiton A, Lewis GF, Adeli K (2000). Mechanisms of hepatic very low density lipoprotein overproduction in insulin resistance. Evidence for enhanced lipoprotein assembly, reduced intracellular ApoB degradation, and increased microsomal triglyceride transfer protein in a fructose-fed hamster model. J Biol Chem.

[B101] Taghibiglou C, Rashid-Kolvear F, Van Iderstine SC, Le-Tien H, Fantus IG, Lewis GF, Adeli K (2002). Hepatic very low density lipoprotein-ApoB overproduction is associated with attenuated hepatic insulin signaling and overexpression of protein-tyrosine phosphatase 1B in a fructose-fed hamster model of insulin resistance. J Biol Chem.

[B102] Kelley GL, Allan G, Azhar S (2004). High dietary fructose induces a hepatic stress response resulting in cholesterol and lipid dysregulation. Endocrinology.

[B103] Zammit VA, Waterman IJ, Topping D, McKay G (2001). Insulin stimulation of hepatic triacylglycerol secretion and the etiology of insulin resistance. J Nutr.

[B104] Park OJ, Cesar D, Faix D, Wu K, Shackleton CH, Hellerstein MK (1992). Mechanisms of fructose-induced hypertriglyceridaemia in the rat. Activation of hepatic pyruvate dehydrogenase through inhibition of pyruvate dehydrogenase kinase. Biochem J.

[B105] Carmona A, Freedland RA (1989). Comparison among the lipogenic potential of various substrates in rat hepatocytes: the differential effects of fructose-containing diets on hepatic lipogenesis. J Nutr.

[B106] Swanson JE, Laine DC, Thomas W, Bantle JP (1992). Metabolic effects of dietary fructose in healthy subjects. Am J Clin Nutr.

[B107] Ostos MA, Recalde D, Baroukh N, Callejo A, Rouis M, Castro G, Zakin MM (2002). Fructose intake increases hyperlipidemia and modifies apolipoprotein expression in apolipoprotein AI-CIII-AIV transgenic mice. J Nutr.

[B108] Noguchi T, Tanaka T (1995). Insulin resistance in obesity and its molecular control. Obes Res.

[B109] Busserolles J, Gueux E, Rock E, Mazur A, Rayssiguier Y (2002). Substituting honey for refined carbohydrates protects rats from hypertriglyceridemic and prooxidative effects of fructose. J Nutr.

[B110] Busserolles J, Gueux E, Rock E, Demigne C, Mazur A, Rayssiguier Y (2003). Oligofructose protects against the hypertriglyceridemic and pro-oxidative effects of a high fructose diet in rats. J Nutr.

[B111] Thirunavukkarasu V, Anuradha CV (2004). Influence of alpha-lipoic acid on lipid peroxidation and antioxidant defence system in blood of insulin-resistant rats. Diabetes Obes Metab.

[B112] Thirunavukkarasu V, Anitha Nandhini AT, Anuradha CV (2004). Effect of alpha-lipoic acid on lipid profile in rats fed a high-fructose diet. Exp Diabesity Res.

[B113] den Boer M, Voshol PJ, Kuipers F, Havekes LM, Romijn JA (2004). Hepatic steatosis: a mediator of the metabolic syndrome. Lessons from animal models. Arterioscler Thromb Vasc Biol.

[B114] Gordon DA, Jamil H (2000). Progress towards understanding the role of microsomal triglyceride transfer protein in apolipoprotein-B lipoprotein assembly. Biochim Biophys Acta.

[B115] Lin MC, Gordon D, Wetterau JR (1995). Microsomal triglyceride transfer protein (MTP) regulation in HepG2 cells: insulin negatively regulates MTP gene expression. J Lipid Res.

[B116] Sato R, Miyamoto W, Inoue J, Terada T, Imanaka T, Maeda M (1999). Sterol regulatory element-binding protein negatively regulates microsomal triglyceride transfer protein gene transcription. J Biol Chem.

[B117] Bartels ED, Lauritsen M, Nielsen LB (2002). Hepatic expression of microsomal triglyceride transfer protein and in vivo secretion of triglyceride-rich lipoproteins are increased in obese diabetic mice. Diabetes.

[B118] Au WS, Kung HF, Lin MC (2003). Regulation of microsomal triglyceride transfer protein gene by insulin in HepG2 cells: roles of MAPKerk and MAPKp38. Diabetes.

[B119] Reaven GM, Chen YD, Jeppesen J, Maheux P, Krauss RM (1993). Insulin resistance and hyperinsulinemia in individuals with small, dense low density lipoprotein particles. J Clin Invest.

[B120] Verschoor L, Chen YD, Reaven EP, Reaven GM (1985). Glucose and fructose feeding lead to alterations in structure and function of very low density lipoproteins. Horm Metab Res.

[B121] Rainwater DL, Mitchell BD, Comuzzie AG, Haffner SM (1999). Relationship of low-density lipoprotein particle size and measures of adiposity. Int J Obes Relat Metab Disord.

[B122] Haidari M, Leung N, Mahbub F, Uffelman KD, Kohen-Avramoglu R, Lewis GF, Adeli K (2002). Fasting and Postprandial Overproduction of Intestinally Derived Lipoproteins in an Animal Model of Insulin Resistance. EVIDENCE THAT CHRONIC FRUCTOSE FEEDING IN THE HAMSTER IS ACCOMPANIED BY ENHANCED INTESTINAL DE NOVO LIPOGENESIS AND ApoB48-CONTAINING LIPOPROTEIN OVERPRODUCTION. J Biol Chem.

[B123] Guo Q, Kohen-Avramoglu R, Adeli K Intestinal assembly and secretion of highly dense/lipid-poor apolipoprotein B48-containing lipoprotein particles in the fasting state: Evidence for induction by insulin resistance and exogenous fatty acids. Metabolism.

